# Gene expression profile of the cartilage tissue spontaneously regenerated *in vivo *by using a novel double-network gel: Comparisons with the normal articular cartilage

**DOI:** 10.1186/1471-2474-12-213

**Published:** 2011-09-29

**Authors:** Ryusei Imabuchi, Yoshihiro Ohmiya, Hyuck Joon Kwon, Shin Onodera, Nobuto Kitamura, Takayuki Kurokawa, Jian Ping Gong, Kazunori Yasuda

**Affiliations:** 1 Department of Sports Medicine and Joint Surgery, Graduate School of Medicine, Hokkaido University, Sapporo, Japan; 2 Bioproduction Research Institute, National Institute of Advanced Industrial Science and Technology, Tsukuba, Japan; 3 Faculty of Advanced Life Science, Hokkaido University, Sapporo, Japan

## Abstract

**Background:**

We have recently found a phenomenon that spontaneous regeneration of a hyaline cartilage-like tissue can be induced in a large osteochondral defect by implanting a double-network (DN) hydrogel plug, which was composed of poly-(2-Acrylamido-2-methylpropanesulfonic acid) and poly-(N, N'-Dimetyl acrylamide), at the bottom of the defect. The purpose of this study was to clarify gene expression profile of the regenerated tissue in comparison with that of the normal articular cartilage.

**Methods:**

We created a cylindrical osteochondral defect in the rabbit femoral grooves. Then, we implanted the DN gel plug at the bottom of the defect. At 2 and 4 weeks after surgery, the regenerated tissue was analyzed using DNA microarray and immunohistochemical examinations.

**Results:**

The gene expression profiles of the regenerated tissues were macroscopically similar to the normal cartilage, but showed some minor differences. The expression degree of COL2A1, COL1A2, COL10A1, DCN, FMOD, SPARC, FLOD2, CHAD, CTGF, and COMP genes was greater in the regenerated tissue than in the normal cartilage. The top 30 genes that expressed 5 times or more in the regenerated tissue as compared with the normal cartilage included type-2 collagen, type-10 collagen, FN, vimentin, COMP, EF1alpha, TFCP2, and GAPDH genes.

**Conclusions:**

The tissue regenerated by using the DN gel was genetically similar but not completely identical to articular cartilage. The genetic data shown in this study are useful for future studies to identify specific genes involved in spontaneous cartilage regeneration.

## Background

Articular (hyaline) cartilage is a highly organized soft tissue [1]. Articular cartilage is frequently damaged due to trauma, and treatment of damaged cartilage is a significant health care concern. It has been a common belief up to now that hyaline cartilage tissue cannot spontaneously regenerate *in vivo *[2,3]. Therefore, the most prevalent strategy to repair the articular cartilage defect is to fill an osteochondral defect with a tissue-engineered cartilage-like tissue or a cell-seeded scaffold material [2,4-6]. However, recent studies have pointed out various practical problems in this strategy, including zoonosis transmission, the need for two-staged surgery, a long period until weight bearing after implantation, an enormous amount of money to establish a therapeutic system [7-11]. Thus, functional repair of articular cartilage defects remains a major challenge in the field of joint surgery and tissue regeneration medicine.

We have paid special attention to the clinical fact that the fibrocartilage tissue can be regenerated in an osteochondral defect by creating many small holes penetrating into the subchondral bone at the bottom of the defect space in order to enhance bleeding from the bone marrow [12]. Namely, the clot formed from bone marrow blood contains mesenchymal stem cells, which can differentiate into cartilage tissues. In addition, recent studies have shown that, in autologous chondrocyte transplantation, quality of the tissue located just beneath the transplanted cells significantly affects quality of the regenerated cartilage [13-15]. In an *ex vivo *study, Engler et al [16] reported that elasticity of the microenvironment in a culture system directs stem-cell differentiation. Therefore, we have considered that, if we implant any bioactive elastic hydrogel at the bottom of an osteochondral defect under conditions similar to in the above-described multiple-penetration surgery, we may be able to induce hyaline cartilage regeneration *in vivo *in the defect space. We have focused on an originally developed PAMPS/PDMAAm double-network (DN) hydrogel [17,18], which was composed of poly-(2-Acrylamido-2-methylpropanesulfonic acid) (PAMPS) and poly-(N, N'-Dimetyl acrylamide) (PDMAAm). In DN gel, the two independently cross-linked polymer networks are physically entangled with each other. The PAMPS network in this DN gel is negatively charged and has a sulphonic acid base, being similar to proteoglycans in normal cartilage. This bioactive DN gel has the elastic modulus of 0.20 MPa [19,20]. In addition, the PAMPS/PDMAAm DN gel surface can enhance differentiation of chondrogenic ATDC5 cells into chondrocytes in the *in vitro *condition [21,22].

Thus, we have recently found a noteworthy phenomenon that, when we implant the PAMPS/PDMAAm DN hydrogel plug at the bottom of an osteochondral defect in the rabbit so that a 2- to 3-mm deep vacant space is intentionally left in the defect, a hyaline cartilage-like tissue rich in type-2 collagen and proteoglycan is spontaneously regenerated *in vivo *in the defect within 4 weeks [21]. Because this phenomenon has a potential that may lead to development of a novel therapeutic method to spontaneously regenerate a hyaline cartilage-like tissue, we should perform multidisciplinary evaluations of the quantity and quality of the regenerated tissue to increase a scientific database of this phenomenon. We have performed histological and immunohistological evaluations [23,24]. However, no biomechanical, biochemical, and genetic studies to evaluate the regenerated tissue have not been reported as of yet. Thus, the present study using DNA microarray has been conducted to genetically clarify what gene expression is induced in the regenerated tissue by the DN gel implantation, and to ask whether the regenerated tissue is genetically identical to the normal articular cartilage. The purpose of the present *in vivo *study using the microarray analysis is to clarify a gene expression profile of the cartilage-like tissue spontaneously regenerated by using the PAMPS/PDMAAm DN gel in comparison with the normal articular cartilage.

## Methods

### 1) Hydrogel preparation

The PAMPS/PDMAAm DN gel was synthesized using the previously reported two-step sequential polymerization method [17]. After polymerization, the PAMPS-PDMAAm DN gel was immersed in pure water for 1 week and the water was changed 2 times every day to remove any un-reacted materials. From the PAMPS/PDMAAm DN gel, we created cylindrical plugs having a 4.5-mm diameter and an 8-mm length.

### 2) Study design

A total of 17 mature female Japanese white rabbits weighing 3.6 ± 0.5 kg were used in this study. Animal experiments were carried out in the Institute of Animal Experimentation, Hokkaido University School of Medicine under the Rules and Regulation of the Animal Care and Use Committee, Hokkaido University School of Medicine.

**Figure 1:**
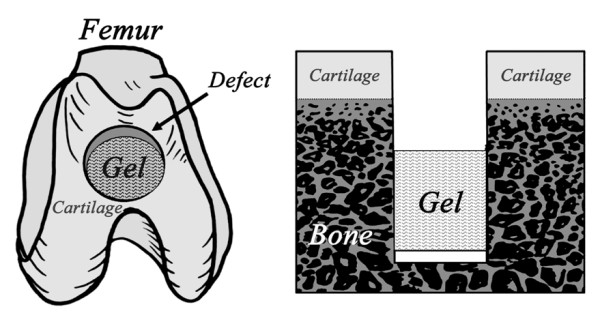
**How to induce cartilage regeneration**. A cylindrical osteochondral defect having a diameter of 4.3-mm was created in the femoral groove. Then, a DN gel plug was implanted into the defect so that a defect having approximately 2-mm depth from the articular surface remained in place after surgery.

Fourteen of the 17 rabbits underwent the previously reported operation [21] in the bilateral knees under intravenous anesthesia (pentobarbital, 25 mg/kg) and sterile conditions. Briefly, we created a cylindrical osteochondral defect having a diameter of 4.3-mm and a depth of 10.0 mm in the femoral groove of the patellofemoral joint, using a drill. Then, we implanted the DN gel plug into the defect so that a defect having approximately 2-mm depth from the articular surface remained in place after surgery (Figure 1). The depth of 2 mm was chosen because this depth was the most effective to induce the spontaneous cartilage regeneration in our previous preliminary study [21]. The incised joint capsule and the skin wound were closed in layers with 3-0 nylon sutures, and an antiseptic spray dressing was applied. Postoperatively, each animal was allowed unrestricted activity in a cage (310 × 550 × 320 mm) without any joint immobilization. Seven animals were sacrificed at 2 and 4 weeks after surgery, respectively. At each period, 5 rabbits were used to analyze a gene profile. The tissue regenerated in the defect was harvested from the 5 rabbits with a surgical knife and a sharp cullet, and mixed into a tissue sample. From the tissue sample, mRNAs were then extracted. In the remaining 2 rabbits, a distal portion of the femur was resected and fixed in a 10% neutral buffered formalin solution. Then, the same histological and immunohistochemical examinations as reported in our previous study [21] were performed to confirm the expression of proteoglycans and type-2 collagen in the cells and the matrix. On the other hand, using the remaining 3 untreated mature rabbits, the normal articular cartilage was harvested from the bilateral femurs in the same manner. Gene expression profiles in the tissues regenerated at 2 and 4 weeks were analyzed with use of custom-made DNA microarray, and compared with a profile of the normal cartilage. The microarray analyses were repeated 3 times using the same mRNA sample to confirm the reproducibility.

### 3) Analysis with microarray

#### RNA isolation and labelling

Total RNA was isolated using the RNA isolation kit (Qiagen Inc., MD, USA), according to the manufacturer's instructions. After monitoring the stability and quantity of the RNA with a 2100 Bioanalyzer (Agilent Technologies, CA, USA), fluorescence-labelled cDNA was synthesized by direct incorporation of Cy3-dUTP or Cy5-dUTP (GE Healthcare Bio-science, Little Chalfont, UK) during random hexamer-primed reverse transcription, using 20 μg of total RNA and the CyScribe First-strand cDNA labelling kit (GE Healthcare Bio-Science).

#### Hybridization and signal detection

Custom oligonucleotide microarrays were obtained from Combimatrix Corp. (Mukilteo, WA, USA). The probes were designed to detect the directly labelled mRNA from 8697 genes which contain 2153 genes from EST library constructed from cartilage tissue of Japanese white rabbits [25] and 6544 genes from the NCBI database: data for some of the genes could not be used in the microarrays, because of their short open reading frames. The probes were loaded on a microarray at least in triplicate.

The microarray was pre-hybridized for 1 hour at 42°C in a solution containing 5 × SSC, 0.1% SDS, 400 mg ml^-1 ^bovine serum albumin, and 100 ng μl^-1 ^salmon sperm DNA. After being washed three times with double distilled water for 2 min, the microarray was hybridized with heat-denatured labelled cDNA in 5 × SSC, 0.1% SDS and 10% formamide for 16 h at 42°C. The array was then washed with 5 × SSC and 0.05% SDS for 4 min, with 0.5 × SSC for 1 min, and with 2 × PBS for 4 min, and fluorescent images of the Cy3 and Cy5 dye channels were obtained using a GenePix 4000B (Axon Instruments, CA, USA). The signal intensity of each spot and its local background were determined using an Array-Pro Analyzer (Media Cybernetics, MD, USA). The net intensity was calculated by subtracting the mean intensity of all pixels within the local background area from the mean intensity of all pixels within each spot area.

## Results

### Histological and immunohistological findings of the regenerated tissue

**Figure 2:**
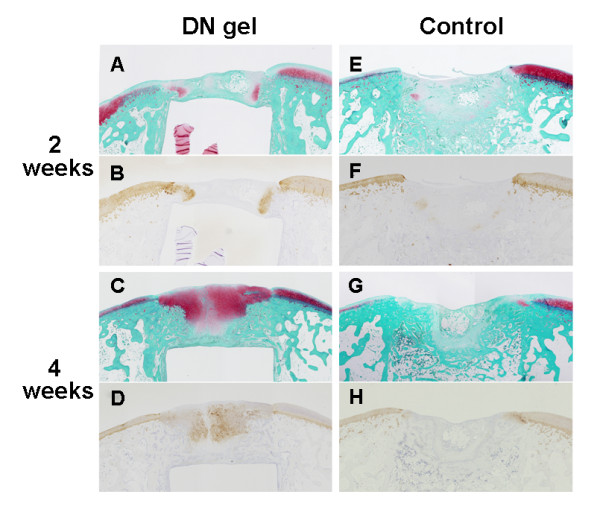
**Regenerated hyaline-cartilage tissues (low magnification, x2)**. A and B: At 2 weeks, symmetrical triangular zones rich in cells were observed in a localized zone close to the bony wall and the implanted gel in the defect. A cartilage-like tissue rich in proteoglycan (A: Safranin-O stain) and type-2-collagen (B: Immunostaining) appeared in the triangular zones. C and D: At 4 weeks, the defect was filled with a sufficient volume of the tissue rich in proteoglycan (C: Safranin-O stain) and type-2-collagen (D: Immunostaining). E, F, G, and H: Control (untreated) specimens at 2 and 4 weeks. The defects were filled with fibrous and osseous tissues.

At 2 weeks, the defect treated with the DN-gel implantation was filled with a blood clot, which was formed by spontaneous bleeding from the bone marrow after surgery. In the blood clot, 2 triangular zones rich in cells were observed close to the bony wall of the defect and the implanted gel. A cartilage-like tissue rich in proteoglycan and type-2-collagen appeared in the triangular zones (Figure 2). At 4 weeks, the defect was filled by a sufficient volume of proteoglycan-rich tissue with regenerated subchondral bone tissue. The immunohistological observation showed that the type-2 collagen was abundantly expressed in the proteoglycan-rich tissue.

### General gene profile of the normal and regenerated tissues

Of the 8697 probe sets arrayed on the DNA chips, the number of genes which showed the expression level greater than 10^-3 ^of that of the greatest expressed gene was 8488 in the normal cartilage, while it was 8479 and 8625 in the regenerated cartilage harvested at 2 and 4 weeks, respectively. These genes were used for the following analyses. These genes were functionally classified into 7 categories based on their biological roles, using the application AmiGO (http://www.godatabase.org). Table 1 shows the classification of the most highly expressed 300 genes in the normal cartilage and the regenerated tissues harvested at 2 and 4 weeks. The rate of each category was completely identical between the regenerated tissues harvested at 2 and 4 weeks. The classification profile of the regenerated tissues was macroscopically similar to that of the normal cartilage, but showed some minor differences (Table 1); e.g., the rate of the cell growth and/or maintenance-related genes was greater (1.7 folds) in the regenerated tissues (12%) than in the normal cartilage (7%). Table 2 shows a list of the most highly expressed 30 genes in the normal cartilage and the regenerated tissues. Not only type-2 collagen-related genes but also Matrix Gla protein (MGP), Signalosome subunit 6 (SN6), and Pleckstrin homology domain (PH domain) genes were seen in each list. On the other hand, Transcription Factor CP2 (TFCP2) and Cbp/p300-interacting Transactivator (CITED) genes were found only in the list on the regenerated tissues (Table 2).

**Table 1:** Functional classification of the most highly expressed 300 genes in the normal cartilage and the regenerated tissues harvested at 2 and 4 weeks.

Functional	Normal	Regenerated tissue	Regenerated tissue
Categories	Cartilage	(2 weeks)	(4 weeks)
Protein metabolism	42 (14.0%)	36 (12.0%)	37 (12.3%)
Cell communication/Signal transduction	25 (8.3%)	24 (8.0%)	23 (7.7%)
Cell growth and/or maintenance	21 (7.0%)	36 (12.0%)	37 (12.3%)
Regulation of nucleobase	14 (4.7%)	16 (5.3%)	15 (5.0%)
Energy pathways/Metabolism	14 (4.7%)	11 (3.7%)	13 (4.3%)
Immune response	12 (4.0%)	9 (3.0%)	10 (3.3%)
Transport	8 (2.6%)	8 (2.7%)	10 (3.3%)
Unknown	164 (54.7%)	160 (53.3%)	155 (51.8%)

(Unit: genes)

**Table 2:** A list of known genes in the top 30 genes most highly expressed in the normal cartilage and the regenerated tissues harvested at 2 and 4 weeks.

Tissue	Place	Gene
Regenerated (2 Wks.)	2	Pro alpha1 type II collagen
	3	Signalosome subunit 6
	4	Luteinizing hormone beta polypeptide
	6	Pro alpha1 type II collagen
	8	COL3A1 protein isoform 9
	12	Proteoglycan 4
	14	Peroxisome proliferator-activated receptor delta (PPARD)
	18	Pleckstrin homology domain containing, family A member 4
	21	Transcription factor CP2
	22	Cbp/p300-interacting transactivator
	23	Type II Collagen
	27	COL3A1 protein, transcript variant 3
	28	Matrix Gla protein

Regenerated (4 Wks.)	1	Luteinizing hormone beta polypeptide
	2	COL3A1 protein isoform 9
	3	Pro alpha1 type II collagen
	5	Pro alpha1 type II collagen
	6	Signalosome subunit 6
	13	Type II Collagen
	14	Cbp/p300-interacting transactivator
	15	Transcription factor CP2
	16	Proteoglycan 4
	19	Matrix Gla protein
	20	COL3A1 protein, transcript variant 3
	22	Matrix Gla protein
	23	Tetraspan NET-6

Normal cartilage	1	T-cell Receptor gamma chain V1.1
	2	Mindbomb homolog 2
	3	Signalosome subunit 6
	10	Pleckstrin homology domain containing, family A
	15	Matrix Gla protein
	20	Spectrin, beta, non-erythrocytic 2
	21	Glutathione synthetase
	22	Peroxisome proliferator-activated receptor delta
	23	Brain protein I3
	29	Pro alpha1 type II collagen
	30	Immunoglobulin heavy chain VDJC-alpha (IgA)

### Cartilage-specific gene profile of the normal and regenerated tissues

#### Collagen-related genes

**Figure 3:**
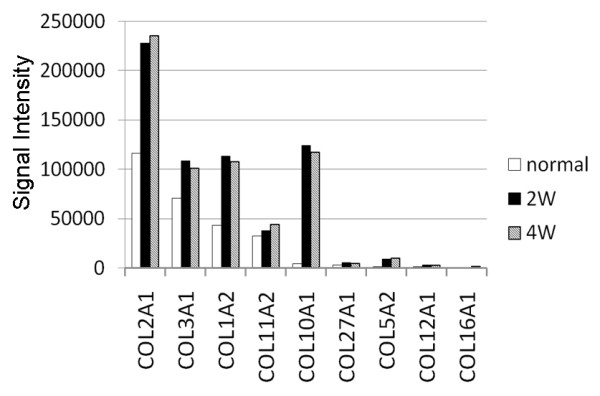
**Expression of collagen-related genes**. In the regenerated tissues, the same genes were dominantly expressed at each period, while the expression degree of collagen (COL) type-2A1, 1A2, and 10A1 genes was obviously greater (twice or more) in comparison with the normal cartilage.

In the normal cartilage, collagen genes of type-2A1, 3A1, 1A2, 11A2, and 10A1 (COL2A1, COL3A1, COL1A2, COL11A2, and COL10A1) were dominantly expressed (Figure 3). In the regenerated tissues, the same genes were dominantly expressed at each period. The expression degrees of COL2A1, COL1A2, and COL10A1 genes in the regenerated tissues were twice or more as large as those in the normal cartilage.

#### Proteoglycan-related genes

**Figure 4:**
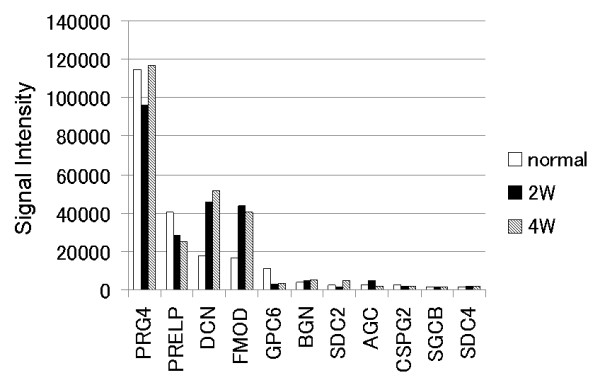
**Expression of proteoglycan-related genes**. In the regenerated tissues, the same genes were dominantly expressed at each period, while the expression degree of DCN and FMOD genes was obviously greater (twice or more) in comparison with the normal cartilage, and the GPC6 gene was expressed to a lesser degrees (one-fourth). (PRG4: proteoglycan 4; PRELP: proline-arginine-rich end leucine-rich repeat protein; DCN: decorin; FMOD: fibromodulin; GPC6: glypican 6; BGN: biglycan; SDC2: syndecan 2; AGC: aggrecan; CSPG2: chondroitin sulfate proteoglycan 2; SGCB: sarcoglycan beta; SDC4: syndecan 4).

In the normal cartilage, proteoglycan 4 (PRG4), proline-arginine-rich end leucine-rich repeat protein (PRELP), decorin (DCN), fibromodulin (FMOD), glypican 6 (GPC6), biglycan (BGN), syndecan 2 (SDC2), and aggrecan genes were dominantly expressed (Figure 4). In the regenerated tissues, the same genes were dominantly expressed at each period. The expression degrees of DCN and FMOD genes in the regenerated tissues were twice or more as large as those in the normal cartilage, and the expression degree of GPC6 gene in the regenerated tissues was quarter as little as those in the normal cartilage.

#### Genes related to noncollagen/nonproteoglycan constituents

**Figure 5:**
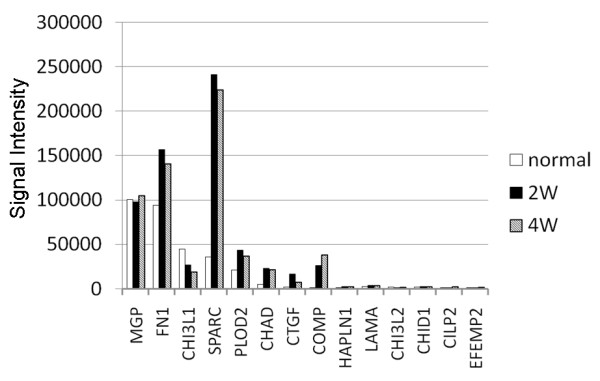
**Expression of genes of noncollagen/nonproteoglycan constituents**. In the regenerated tissues, the same genes were dominantly expressed at each period, while the expression degree of SPARC, FLOD2, CHAD, CTGF, and COMPs genes was obviously greater (twice or more) in comparison with the normal cartilage. (MGP: matrix G protein; FN1: fibronectin 1; CH13L1: chitinase 3-like 1; SPARC: secreted protein acidic and rich in cysteine; procollagen-lysine; PLOD2: procollagen-lysine 2-oxoglutarate 5-dioxygenase 2; CHAD: chondroadherin; CTGF: cartilage transformation growth factor; COMP: cartilage oligomeric matrix protein; HAPLN1: hyaluronan and proteoglycan link protein 1; LAMA: Laminin subunit alpha-4; CH13L2: chitinase 3-like 2: CHID1: chitinase 1; CILP2: cartilage intermediate layer protein 2; EFEMP2: EGF-containing fibulin-like extracellular matrix protein 2).

In the normal cartilage, MGP, fibronectin 1 (FN1), chitinase 3-like 1 (CH13L1), secreted protein acidic and rich in cysteine (SPARC), procollagen-lysine 2-oxoglutarate 5-dioxygenase 2 (PLOD2), chondroadherin (CHAD), cartilage transformation growth factor (CTGF), and cartilage oligomeric matrix proteins (COMPs) genes were dominantly expressed (Figure 5). In the regenerated tissues, the same genes were dominantly expressed at each period. The expression degrees of SPARC, FLOD2, CHAD, CTGF, and COMPs genes in the regenerated tissues were twice or more as large as those in the normal cartilage.

### Histological and immunohistological findings of the regenerated tissue

We determined the relative expression level of each gene in the regenerated tissues to the normal cartilage. At 2 and 4 weeks, 307 and 270 genes, respectively, were expressed 5 times or more as compared with the normal cartilage. The functional classification profiles of these genes were identical between the 2 periods (Table 3). Major categories were comprised of protein metabolism-related genes (26%), cell growth- and maintenance-related genes (20-21%), and cell communication/signal transduction-related genes (10-11%). Minor categories (2-5%) included transport-related genes, energy pathway-related genes, nucleobase regulation-related genes, and immune response-related genes. Functions of 30% of the genes were unclassified. Table 4 shows a list of the top 30 genes that were expressed 5 times or more as compared with the normal cartilage. The list includes type-2 and type-10 collagen genes, which are related to matrix collagen, as well as FN, vimentin, and COMP genes, which are related to noncollagen/nonproteoglycan constituents of the extracellular matrix. The list also involves elongation factor 1 alpha (EF1alpha) and TFCP2 genes, which are classified as regulation-related genes, as well as glyceraldehyde-3-phosphate dehydrogenase (GAPDH) gene, which is categolized as energy metabolism-related gene (Table 4).

**Table 3:** Functional classification of 307 and 270 genes that were expressed in the regenerated tissues harvested at 2 and 4 weeks, respectively, 5 times or more as compared with the normal cartilage.

Functional categories	2 weeks	4 weeks
Protein metabolism	79 (25.7%)	69 (25.7%)
Cell growth and/or maintenance	63 (20.5%)	54 (20.0%)
Cell communication/Signal transduction	32 (10.4%)	29 (10.7%)
Transport	14 (4.6%)	13 (4.8%)
Energy pathways/Metabolism	12 (3.9%)	9 (3.3%)
Regulation of nucleobase	10 (3.3%)	9 (3.3%)
Immune response	5 (1.6%)	6 (2.2%)
Unknown	92 (30.0%)	81 (30.0%)

(Unit: genes)

**Table 4:** A list of the top 30 genes that were expressed in the regeneration tissues harvested at 2 or 4 weeks 5 times or more as compared with the normal cartilage.

Place	2W	4W
1	elongation factor 1 alpha	elongation factor 1 alpha
2	osteonectin	glyceraldehyde-3-phosphate dehydrogenase
3	glyceraldehyde-3-phosphate dehydrogenase	osteonectin
4	collagen type X	collagen type X
5	collagen type X	transcription factor CP2
6	transcription factor CP2	collagen type X
7	cystatin C	cystatin C
8	collagen type III alpha 1	collagen type III alpha 1
9	unknown	osteonectin
10	osteonectin	osteonectin
11	vimentin	unknowm
12	osteonectin	unknowm
13	vimentin	C-type lectin superfamily member 1
14	unknown	vimentin
15	ubiquitin B	hemoglobin, beta
16	unknown	COMP
17	C-type lectin superfamily member 1	Vimentin
18	fibronectin	ribosomal protein L17
19	tartrate-resistant acid phoshatase	COMP
20	ribosomal protein S8	unknowm
21	ribosomal protein L17	ubiquitin B
22	unknown	fibronectin
23	cartilage oligomeric matrix protein	ribosomal protein S8
24	ribosomal protein L17	osteonectin
25	collagen type X	collagen type X
26	pP47 protein	decorin
27	unknown	ribosomal protein L17
28	23 kD highly basic protein	ribosomal protein S18
29	ribosomal protein S18	unknowm
30	osteonectin	Ribosomal protein L18A

In addition, 145 and 121 genes were expressed one fifth or less as compared with the normal cartilage at 2 and 4 weeks, respectively. The functional classification profiles of the 145 and 121 genes were found to be almost similar (Table 5): The rate of each category was less than 10%, while functions of 63-66% of the genes were unclassified. The bottom 30 genes which were expressed in the regenerated tissues one fifth or less as compared with the normal cartilage at each period were listed in Table 6.

**Table 5:** Functional classification of 145 and 121 genes that were expressed in the regenerated tissues harvested at 2 and 4 weeks, respectively, one fifth or less as compared with the normal cartilage.

Functional categories	2 weeks	4 weeks
Cell communication/Signal transduction	12 (8.3%)	12 (9.9%)
Energy pathways/Metabolism	11 (7.6%)	8 (6.6%)
Immune response	11 (7.6%)	8 (6.6%)
Regulation of nucleobase	8 (5.5%)	8 (6.6%)
Transport	4 (2.7%)	4 (3.3%)
Protein metabolism	2 (1.4%)	3 (2.5%)
Cell growth and/or maintenance	2 (1.4%)	1 (0.8%)
Unknown	95 (65.5%)	77 (63.7%)

(Unit: genes)

**Table 6:** A list of the bottom 30 genes that were expressed in the regeneration tissues harvested at 2 or 4 weeks one fifth or less as compared with the normal cartilage at each period.

Place	2W	4W
1	Clusterin	translokin
2	unknown	stromal interaction molecule 2
3	Unknown	Lactase phlorizin hydrolase
4	5-hydroxytryptamine (serotonin) receptor 7	unknown
5	Lactase phlorizin hydrolase	unknown
6	N207 immunoglobulin mu heavy chain VDJ region	unknown
7	translokin	CG32425-PA
8	NFI-B	N207 immunoglobulin mu heavy chain VDJ region
9	unknown	unknown
10	unknown	unknown
11	unknown	unknown
12	unknown	clusterin
13	unknown	CGMP-dependent protein kinase I
14	Chloride channel	unknown
15	zinc finger protein 282	NFI-B
16	unknown	unknown
17	upregulated during skeletal muscle growth 5	RAG-2
18	PiUS	zinc finger protein 282
19	stromal interaction molecule 2	heat shock 70 kDa protein 5
20	unknown	unknown
21	Myosin light chain kinase 2	PiUS
22	unknown	calnexin
23	nudix -type motif 9	G-protein coupled receptor 48
24	TNF superfamily, member 2	P235 immunoglobulin mu heavy chain VDJ region
25	unknown	Myosin light chain kinase 2
26	ceruloplasmin	Proteolipid protein
27	unknown	unknown
28	unknown	unknown
29	unknown	unknown
30	mitochondrial trifunctional protein, beta subunit	mitochondrial trifunctional protein, beta subunit

## Discussion

This study clarified a gene expression profile of the cartilage-like tissue regenerated by using the PAMPS/PDMAAm DN gel in comparison with that of the normal mature cartilage. The present study showed that gene expression profiles of the tissues spontaneously regenerated at both 2 and 4 weeks by using the PAMPS/PDMAAm DN gel were similar to the normal cartilage. Type-2 collagen-related genes, MGP, SN6, and PH domain genes were highly expressed in both the tissues. Type-2 collagen-related genes are the most essential markers that upregulate upon hyaline cartilage differentiation, and MGP is a calcification inhibitor in the cartilage [26]. SN6 and PH domain genes were highly expressed within all the tissues. Roles of these molecules in chondrogenesis have been unknown. However, we should note that mammalian constitutive photomorphogenesis 9 SN6 connects signaling with the ubiquitin-mediated proteasome degradation pathway and is implicated in cell cycle regulation and DNA damage response [27], and that PH domains play a role in recruiting proteins to different membranes and enable proteins to interact with other components of the signal transduction pathways [28]. Therefore, the present study suggested that the regenerated cells were genetically differentiated into the hyaline cartilage cells by 2 weeks. Histological and immunohistochemical examinations in the present study showed that type-2 collagen and proteoglycan molecules were highly expressed in the regenerated cells and matrix at 2 and 4 weeks, respectively. These results were supported by our previous study [21]. Additionally, we took notice of the immune-related genes in Tables 1 and 3. The ratio of immune-related gene expression was identical among the normal cartilage and the regeneration tissues. These facts implied that the implantation of the PAMPS/PDMAAm DN gel plug did not induce any immune reactions in the rabbit, although it was a foreign body.

Secondly, the present study demonstrated that the cartilage marker gene profile of the tissues regenerated by the DN gel implantation was similar to that of the normal cartilage, concerning the types of highly expressed genes. Genetically, we can regard the regenerated tissue as the hyaline cartilage. However, the gene profile of the regenerated tissue has some differences concerning the quantity of each expression level. In the regenerated tissues, the expression degree of COL2A1, COL1A2, and COL10A1 genes in the genes encoding collagens, DCN and FMOD genes in the genes encoding proteoglycans, and SPARC, FLOD2, CHAD, CTGF, and COMPs genes in the genes encoding noncollagen/nonproteoglycan constituents of the extracellular matrix were obviously greater in comparison with the normal cartilage. COL10A1 is an important marker of prehypertrophic and hypertrophic chondrocytes. DCN is produced predominantly by mesenchymal stem cells, binds to type-2 collagen, and is involved in the control of fibrillogenesis [29]. FMOD interacts with type-1 and type-2 collagen fibrils and inhibits fibrillogenesis *in vitro*, and may participate in the assembly of the extracellular matrix *in vivo *[30]. SPARC modulates synthesis as well as turnover of the collagenous extracellular matrix. [31]. PLOD2 is regulated with total collagen synthesis [32]. CHAD binds to two sites on type-2 collagen. Both CHAD and collagen interact with chondrocytes, partly via the same receptor, but give rise to different cellular responses [33]. CTGF is an important growth factor that coordinates chondrogenesis [34]. COMPs are prominent in cartilage; however, it is also present in tendon and binds to type-1 and type-2 collagens with high affinity [35,36], although the functions is not sufficiently clarified. Therefore, the above-described results suggested that various genes concerning condrogenesis were strongly enhanced in the tissues spontaneously regenerated by implanting the DN gel. However, it is unclear whether such gene expression profile is specific characteristics of the cartilage tissue regenerated by the DN gel implantation or common characteristics of cartilage tissues regenerated with tissue-engineering techniques, because gene expression profiles of the tissue-engineered cartilage tissues have not been clarified as of yet.

Table 3 and Table 5 show a category of the genes that were different in the expression level between the normal cartilage and the tissues regenerated by the DN gel implantation. Specifically, we paid attention to the fact that protein metabolism-related genes of more than 60 (26%) and cell growth-related genes of more than 40 (20%) were highly expressed 5 times or more than the normal articular cartilage in the regenerated cartilage tissue. We carefully checked these up-regulated genes, and did not find any genes related to tumor or abnormal cell growth, such as alpha-fetoprotein, beta-2-microglobulin, beta-HCG, Bladder tumor antigen, CA15-3, CA19-9, CA27, CA29, CA72-4, CA125, calcitonin, carcinoembryonic antigen, chromogranin A, epidermal growth factor receptor, hormone receptors, HER2, human chorionic gonadotropin, immunoglobulins, neuron-specific enolase, NMP22, prostate-specific antigen, prostatic acid phosphatase, prostate-specific membrane antigen, S-100, TA-90, thyroglobulin, and etc. These results indicated that the genes up-regulated in the present study were not related to differentiation to tumor cells, but to the chondrocyte itself. In addition, the gene profile shown in the present study suggested that the regeneration process continued over the 4 week period. In the histological findings, we observed a little unrepaired lesion in the middle of the regenerated cartilage. The 4 week period after implantation is considered to be insufficient to complete a comprehensive osteochondral regeneration in adult rabbit knees with a full-thickness defect. This information will contribute to an increase of basic database to create an effective protocol for regeneration of cartilage in the near future.

In the present study, we found that TFCP2, CITED, and elongation factor-1 (EF-1) alpha were highly expressed in the cartilage tissue regenerated by the DN gel implantation in comparison to the normal cartilage. It is known that TFCP2 regulates genes involved in development of mesenchymal stem cells [37] and CITED controls the transcription activity of Cart1 involved in skeletal development [38]. Therefore, we speculate that TFCP2 and CITED may play an important role in the cartilage regeneration induced by the DN gel implantation. In addition, it has been reported that EF-1 alpha is related to protect a cell from endoplasmic reticulum stress [39], which reduces both chondrocyte growth and matrix expression and induces chondrocyte apoptosis [40]. Therefore, we speculate that EF-1 alpha also may contribute to the DN gel-induced cartilage regeneration by suppressing endoplasmic reticulum stress.

In the list of the top 30 genes that were expressed in the regenerated tissues 5 times or more as compared with the normal cartilage, we have taken notice of fibronectin and vimentin. It has been known that fibronectin plays a critical role in prechondrogenic condensation in the early stage of chondrogenesis [41] and that vimentin contributes to the maintenance of the chondrocyte phenotype [42]. Therefore, the highy expressed fibronectin and vimentin may contribute to the spontaneous cartilage regeneration by stimulating the condensation process of mesenchymal stem cells and maintaining cartilage phenotype, respectively. In addition, we also found that GAPDH, which plays a significant role in glycolysis, was highly expressed in the regenerated cartilage in comparison with the normal cartilage. It is known that the energy is supplied by glycolysis in a hypoxia environment rather than by mitochondrial respiration [43]. Escoubet et al [44] reported that hypoxia increases GAPDH transcription in rat alveolar epithelial cells. Therefore, the high expression of GAPDH observed in the present study implies that glycolysis is enhanced in the cartilage regeneration induced by the DN gel implantation in order to respond to a high energy demand in a low-oxygen environment.

Finally, the present study has demonstrated that the tissue regenerated *in vivo *by using the PAMPS/PDMAAm DN gel can be genetically regarded as the hyaline cartilage, although it has some minor differences from the normal cartilage. Therefore, we can conclude that spontaneous articular cartilage regeneration can be induced *in vivo *by using the PAMPS/PDMAAm DN gel. However, biomechanical and biochemical studies are needed to develop a novel therapeutic method to induce the spontaneous articular cartilage regeneration. The present study includes a few limitations. First, we did not perform statistical analyses. Secondly, we did not evaluated functions of the highly expressed genes. Further studies are needed to clarify genetic mechanisms of the spontaneously regenerated cartilage. However, the genetic data shown in this study are considered to be useful for future studies to identify specific genes involved in spontaneous cartilage regeneration and to make their mechanisms clear.

## Conclusions

The present study has demonstrated that the tissue spontaneously regenerated *in vivo *by using the PAMPS/PDMAAm DN gel can be genetically regarded as the hyaline cartilage, although it has some minor differences from the normal cartilage. The expression degree of COL2A1, COL1A2, COL10A1, DCN, FMOD, SPARC, FLOD2, CHAD, CTGF, and COMP genes was greater in the regenerated tissue than in the normal cartilage. The top 30 genes that expressed 5 times or more in the regenerated tissue as compared with the normal cartilage included type-2 collagen, type-10 collagen, FN, vimentin, COMP, EF-1 alpha, TFCP2, and GAPDH genes. The genetic data shown in this study are useful for future studies to identify specific genes involved in spontaneous cartilage regeneration and to make their mechanisms clear.

## Competing interests

We have no financial or non- financial competing interests. We do not hold or are not currently applying for any patents relating to the content of the manuscript.

## Authors' contributions

RI performed the animal experiment and the microarray analysis. YO instructed the microarray analysis. HJK instructed isolation of total RNA and synthesis of the fluorescence-labelled cDNA. SO contributed to the data analysis. NK instructed the animal experiment and performed the histological examinations. TK and JPG created the DN-gel material. KY designed the study and drafted the manuscript. All authors read and approved the final manuscript.

## Pre-publication history

The pre-publication history for this paper can be accessed here:

http://www.biomedcentral.com/1471-2474/12/213/prepub
